# Supplementary figures and images for: How caged salmon respond to waves depends on time of day and currents
Source: PeerJ. 2020 Jun 18;8:e9313. doi: 10.7717/peerj.9313 (PMC7306220; doi:10.7717/peerj.9313)

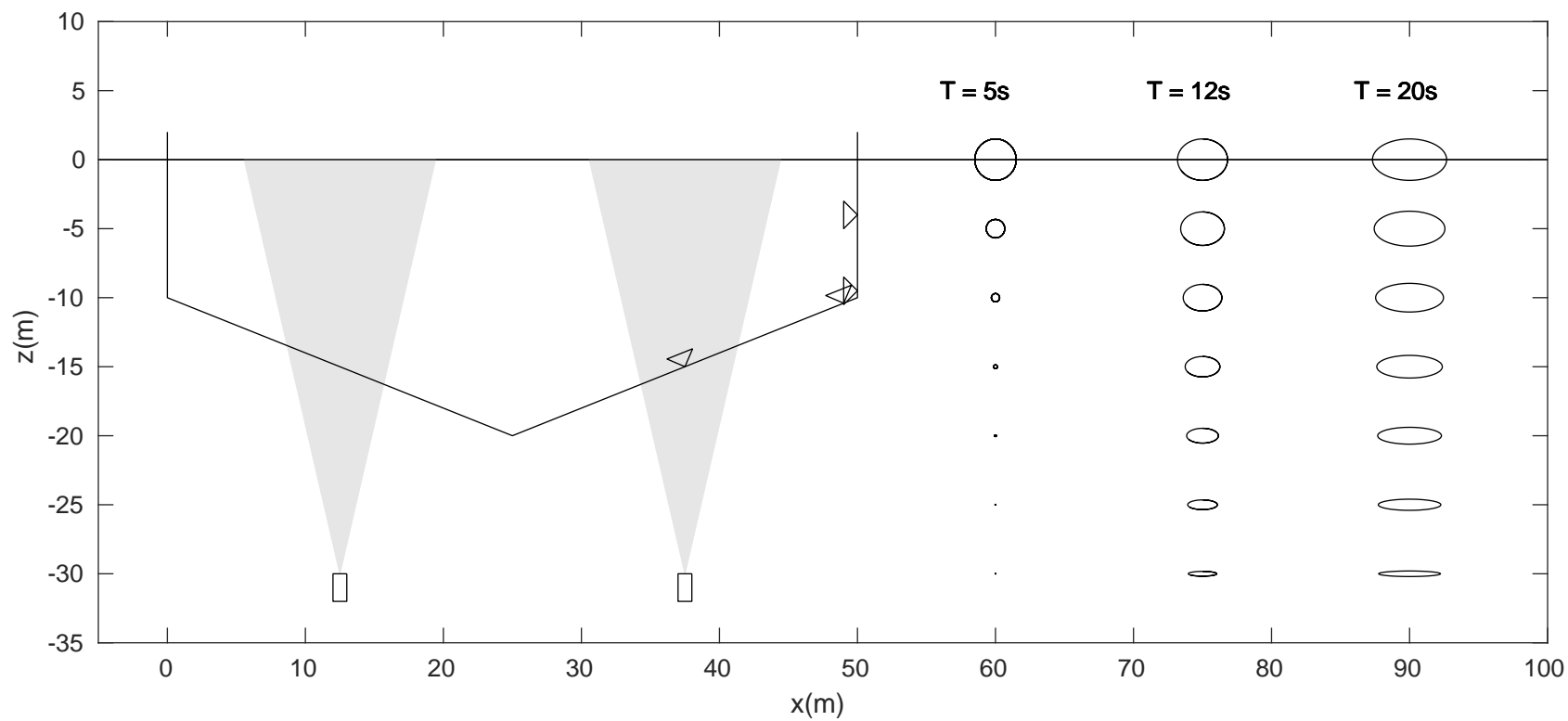

Supplement: Figure S1 — A to scale layout of the salmon cage and recording equipment location. Gray cones illustrate the area covered by the front (right) and back (left) echo sounders. Waves (entering at the front of the cage) with three different wave periods is added to illustrate the relative depth of the cage compared to wave attenuation through the water column. [file peerj-08-9313-s001.pdf]

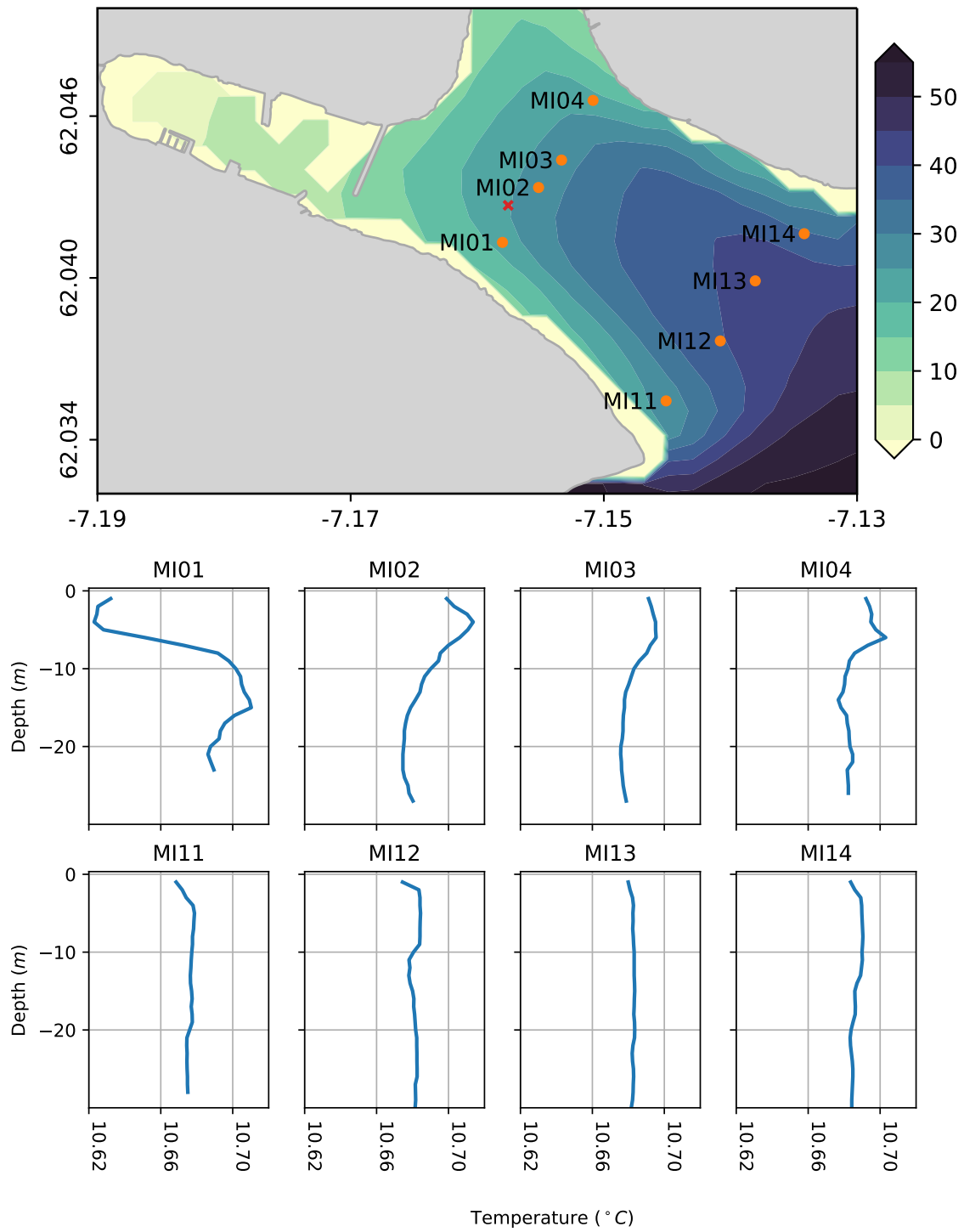

Supplement: Figure S2 — Six panels (top) are temperature profiles measured in the fjord with MI1 being the closest to the farm. Temperature changes less than 0.1 degrees centigrade. At the bottom is a map showing the approximate locations of sampled stations as well as the salmon farm used in this study. [file peerj-08-9313-s002.pdf]

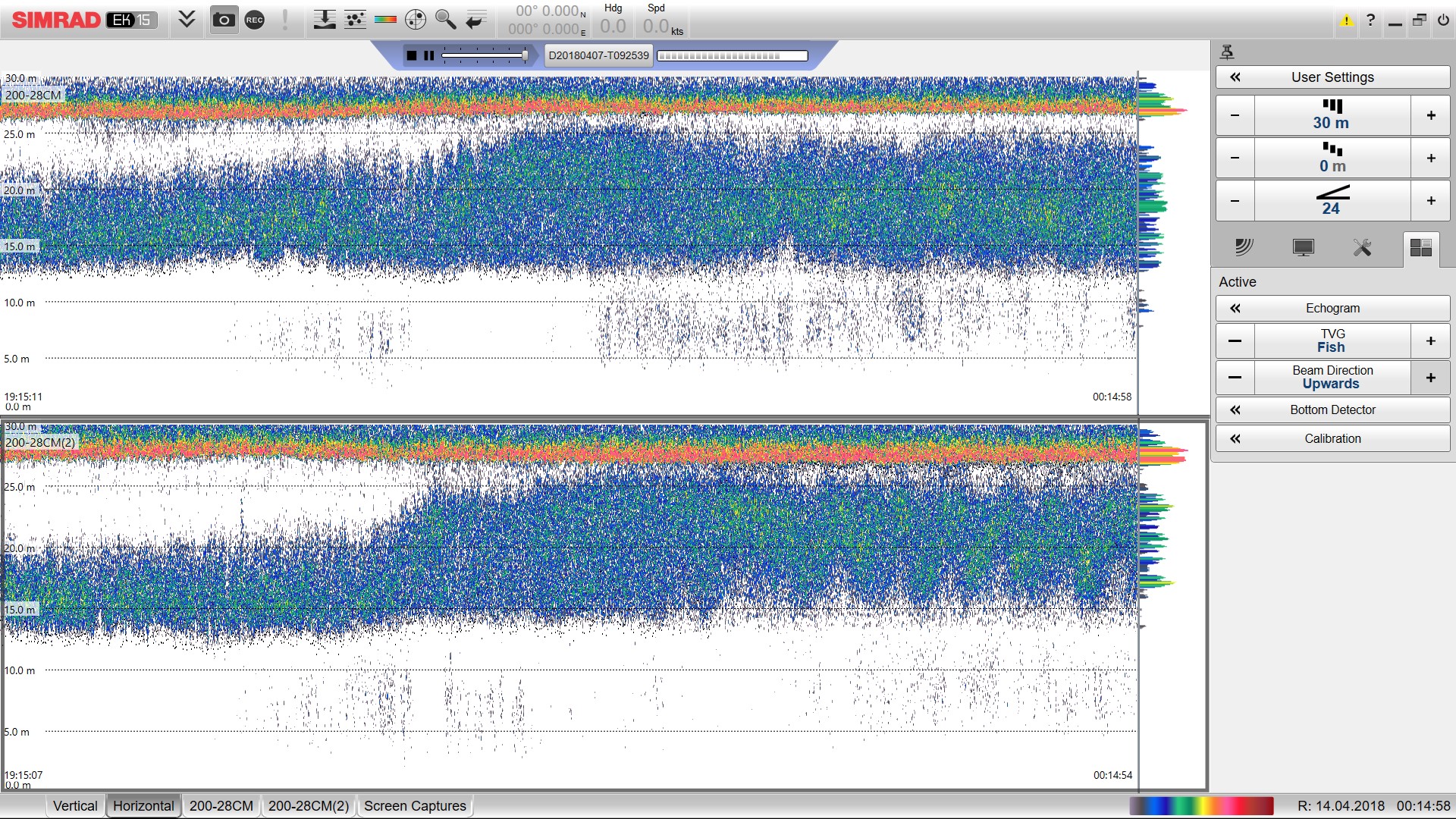

Supplement: Figure S3 — Both echo sounders (top and bottom panel) show a clear surface (orange band) and salmon shoal (blue green mass). The slight change in fish position and vertical distribution coincides with sunset or change from light to dark (the far left is 7PM and the far right is midnight). Some fish or noise can be seen less than 10m away from the echo sounder. As this is outside of the cage, these data were removed. [file peerj-08-9313-s003.jpg]

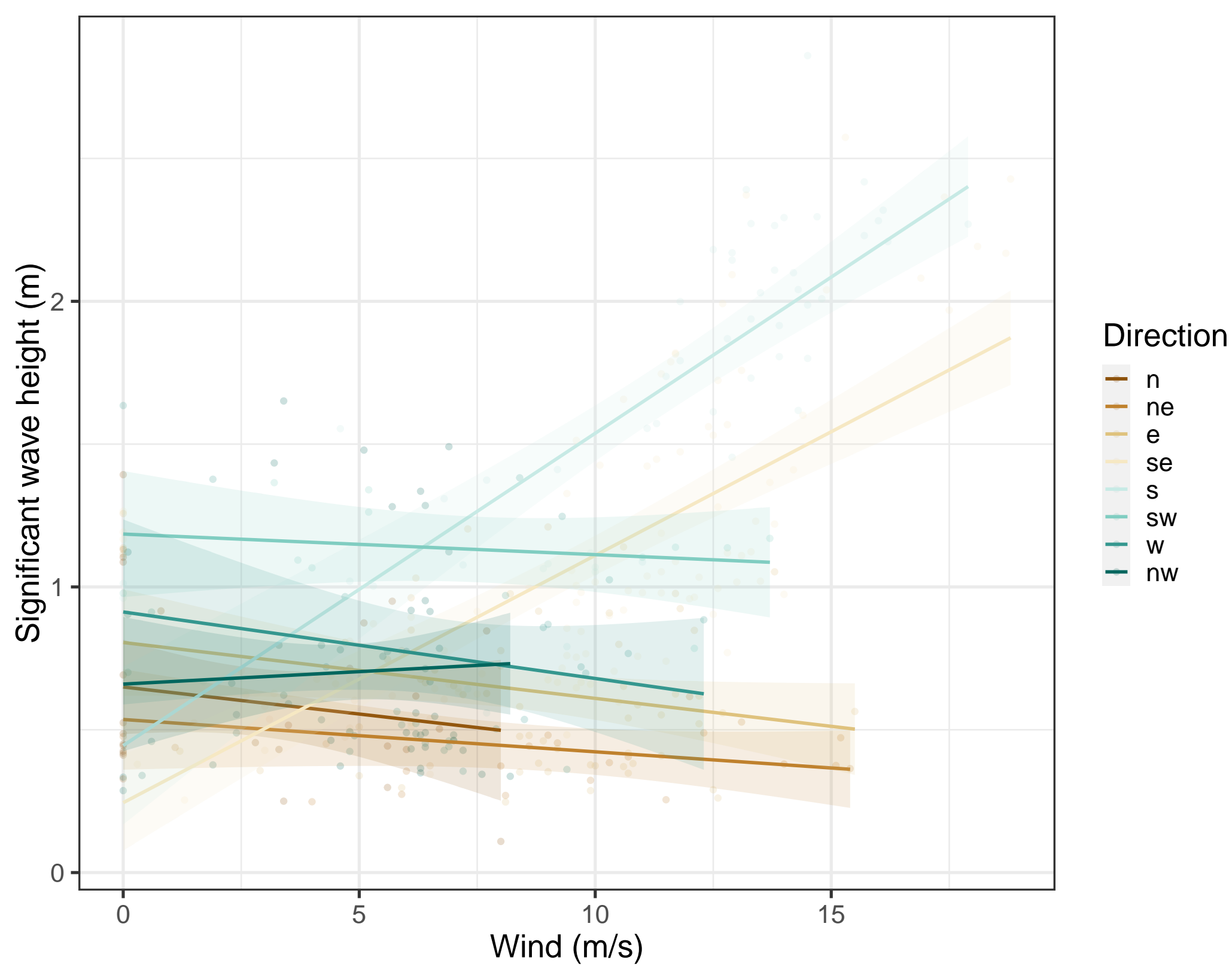

Supplement: Figure S4 — Lines of best fit for each direction with standard error bands. Significant wave height (Hs) correlates positively with wind speed at wind directions between 90 and 180 degrees (”se” = 90–134 and ”s” = 135–179). [file peerj-08-9313-s004.pdf]

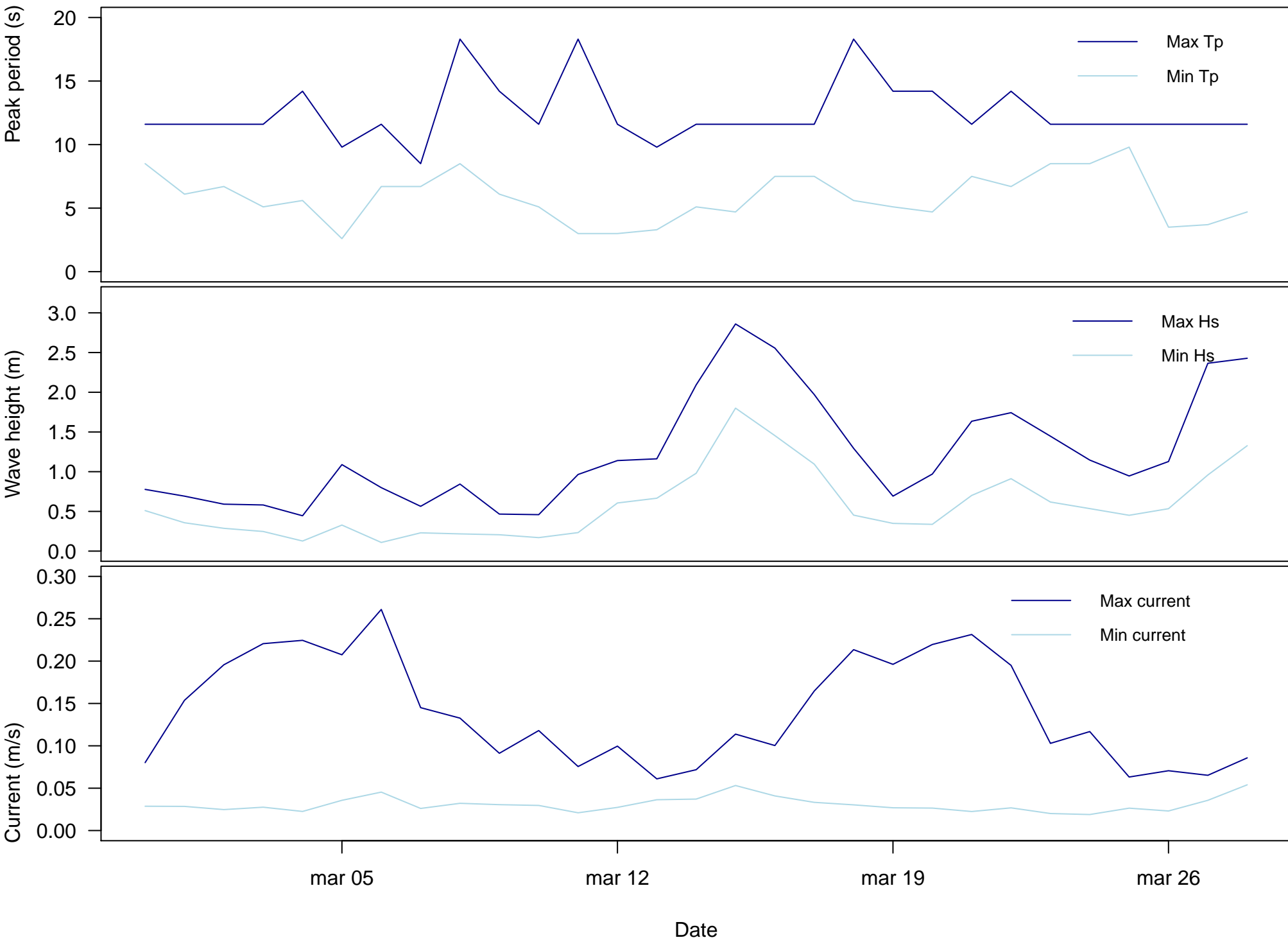

Supplement: Figure S5 — Lines show the highest and lowest values measured each day for Hs, Tp, and current speed respectively. [file peerj-08-9313-s005.pdf]

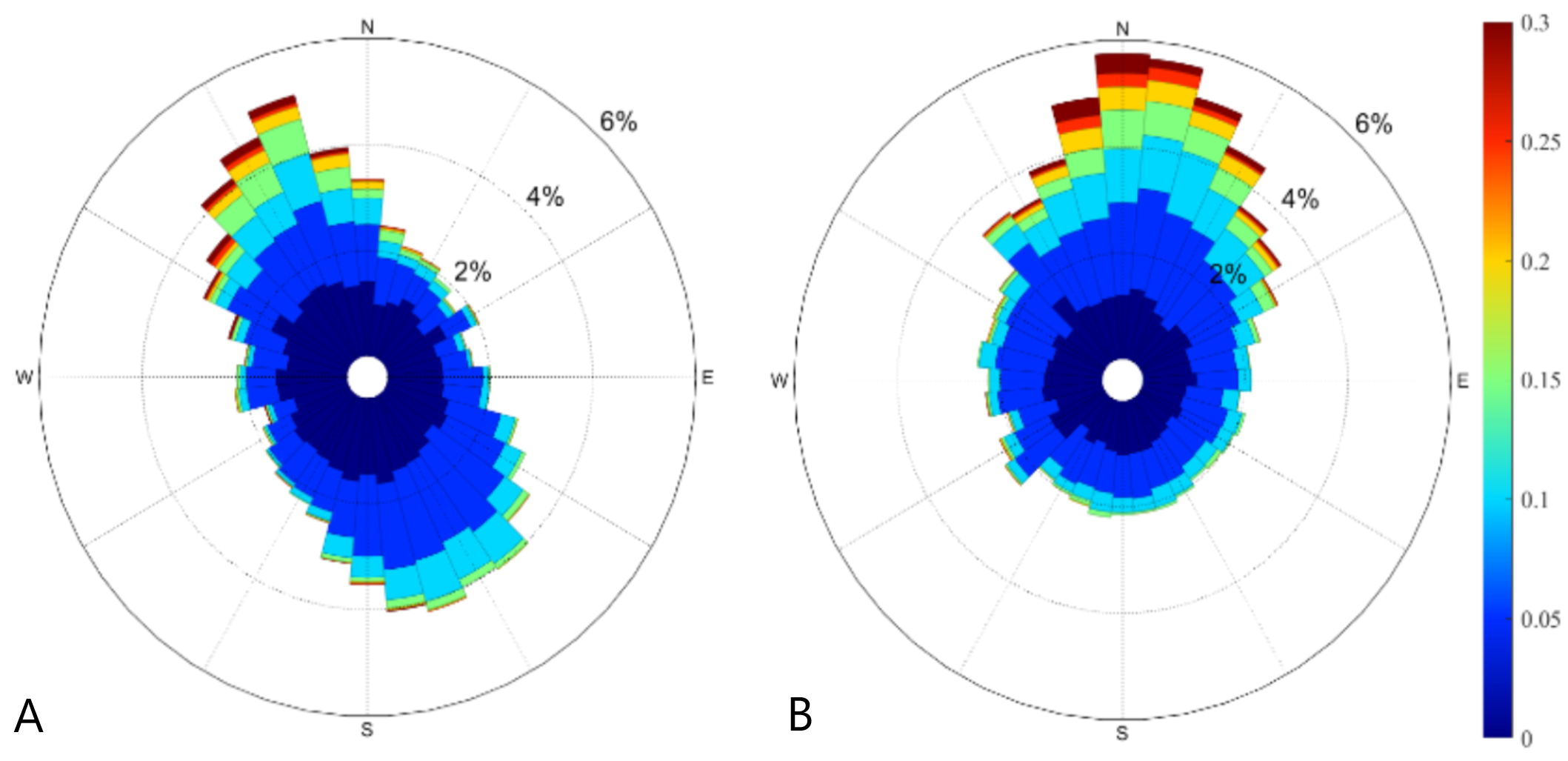

Supplement: Figure S6 — (A) current directions at the south of the cage and (B) current directions at the east of the cage. [file peerj-08-9313-s006.png]
